# New clinical results with sGC stimulators

**DOI:** 10.1186/1471-2210-11-S1-O2

**Published:** 2011-08-01

**Authors:** Jürgen Behr

**Affiliations:** 1 Department of Internal Medicine III, University Hospital Bergmannsheil, Bochum, Germany

## Background

Stimulators of the nitric oxide (NO) receptor soluble guanylate cyclase (sGC) are a new class of substances which enhance the biosynthesis of cyclic guanosin - monophosphate (cGMP) independently from the presence of nitric oxide (NO). In pulmonary smooth muscle cells, increased intracellular cGMP concentrations lead to vasodilatation, making sGC-stimulators potentially useful for the treatment of patients suffering from pulmonary hypertension (PH). Despite recent advances in the management of PH there is still significant morbidity and mortality from this condition which may occur in isolation (idiopathic pulmonary arterial hypertension, iPAH) or in the context of different clinical settings including chronic thromboembolic pulmonary hypertension (CTEPH), PH in chronic obstructive pulmonary diseases (PH-COPD), in interstitial lung disease (PH-ILD) or in left heart disease (PH-LHD).

## Results

Riociguat is an orally active sGC-stimulator that has been shown to have a favourable tolerability profile. Consequently, Riociguat has been tested in small pilot studies and in one phase II clinical trial in PAH and CTEPH [1] as well as in PH-COPD [2] and PH-ILD [3]. The results of these studies suggest that Riociguat is a potential treatment option for all of these conditions by improving pulmonary hemodynamics. For PAH and CTEPH a positive effect on clinical endpoints including the six minute walking distance has also been demonstrated [1]. As a result phase III clinical trials have been launched for PAH (PATENT-trial), CTEPH (CHEST-trial) and are preparation for the other diseases.

## Conclusion

Based on the pharmacologic characteristic and on the preliminary results from phase II studies, Riociguat is a promising new drug which will hopefully be available soon for the treatment of pulmonary hypertension in different patient populations suffering from this condition.
